# The Effect of Pre-operative use of Antiplatelets and Anticoagulants on Time to Surgery in Hip Fracture Patients

**DOI:** 10.5704/MOJ.2303.018

**Published:** 2023-03

**Authors:** NS Shamsuri, CY Yeap, KMS Low, T Kaur-Dhaliwal, H Hashim, AY Wan-Sim, SD Chandrakumara, KSA Yeo, KS Goh

**Affiliations:** 1 Department of Pharmacy, Changi General Hospital, Singapore; 2 Department of Pharmacy, Sengkang Community Hospital, Singapore; 3 Clinical Research Unit, Khoo Teck Puat Hospital, Singapore; 4 Department of Geriatric Medicine, Changi General Hospital, Singapore; 5 Department of Case Management, Changi General Hospital, Singapore; 6 Department of Orthopaedic Surgery, Changi General Hospital, Singapore

**Keywords:** hip fracture, surgical delay, time to surgery, anticoagulant use, antiplatelet use

## Abstract

**Introduction:**

Hip fractures are a major health concern resulting in significant morbidity worldwide. They are the leading cause of fall-related injuries amongst the elderly with high risk of death, and numbers are expected to rise with the growing elderly population. Expedited surgical repair has been proven to improve patient outcomes, however there are often multiple barriers to early surgery especially in the elderly. The use of antiplatelets and anticoagulation is a significant contributory factor to surgical delay.

**Materials and methods:**

We conducted a retrospective, single centre study on hip fracture patients admitted to an acute care orthogeriatric unit over a 12-month period, aimed at determining the impact of pre-operative use of antiplatelets and anticoagulants on time to surgery (TTS) and its impact on one-year mortality rates.

**Results:**

Amongst 404 eligible patients, 102 were on antiplatelets, 23 on anticoagulants and 279 were neither on antiplatelets or anticoagulants. Our study showed that patients taking clopidogrel (p<0.001) and DOACs (p=0.001) were more likely to have delayed surgery compared to those who were not on these agents. In addition, all patients on warfarin experienced surgical delay. Warfarin group also had highest mortality rates compared to other group and 10 times more likely to die within a year (p=0.001).

**Conclusion:**

The results from this study are consistent with existing literature, suggesting that the use of clopidogrel and anticoagulants have a negative impact on TTS in hip fracture patients. Strategies should be developed for patients on these medications to enhance their TTS.

## Introduction

Fragility hip fractures represent a serious injury with high risk of death with approximately 5% of all-cause mortality attributable to hip fractures^1^. The incidence of hip fractures is closely associated with reduced bone mineral density which decreases as one ages. It is estimated that by 2050, the population aged above 70 years will rise to 1.5 million in Singapore and more than 50% of all osteoporotic fractures will occur in Asia^2,3^.

Surgical repair remains the mainstay of treatment of hip fractures. Existing guidelines recommend early surgery in order to preserve function, alleviate pain and improve mobility. The definition of early surgery however remains unclear^4^, with varying guidelines recommending surgery as early as 24 hours or within 48 hours of admission^4-6^. Anaesthesia advocates for surgery within 36 hours of hip fracture^7^. Surgical delay increases risk of immobility-related complications such as pressure sores, deep vein thrombosis, urinary tract infections and pulmonary embolism^8^. However, surgical delays are sometimes warranted for pre-operative optimisation and correction of medical conditions to ensure fitness for surgery^9^. Correspondingly, haematological abnormalities and use of antiplatelets and anticoagulants are common reasons for surgical delay^10^. In the elderly population, presence of multiple comorbidities and cardiovascular conditions often necessitate the use of antiplatelets and anticoagulants. In Singapore, the percentage of patients commenced on anticoagulation for atrial fibrillation (AF) increased from 48% to 61.9% within a year from 2014^11^. With the prevalent use of antiplatelets and anticoagulation, physicians are often faced with a challenge in deciding between proceeding with surgery with a higher risk of bleeding or delaying surgery with accompanying complications^12^.

Antiplatelets have long been studied in hip fracture patients with two most commonly used agents being aspirin and clopidogrel. The inhibitory action of these drugs on platelets is irreversible^13^ but they entail different perioperative management. Aspirin need not be discontinued perioperatively as it has not been proven to increase significant bleeding post-surgery^14^. There are however conflicting views regarding clopidogrel. For neuraxial blockade during surgery, clopidogrel requires at least 5 days of drug washout^15,16^. The main concerns are that of increased bleeding risk and hematoma formation following spinal anaesthesia, though no evidence exists that clopidogrel alone increases the risk of vertebral canal hematoma. Recent guidelines, however, recommends clopidogrel users to proceed with early surgery under general anaesthesia (GA) and to consider spinal anaesthesia if risk with GA is high, after careful discussion with patient^7^.

Perioperative management of anticoagulants involves finding a balance between risks of bleeding and thromboembolism associated with interruption. Reversal of warfarin is often practiced with an aim to reach an INR of <1.5 before proceeding with surgery^15^. However, there is uncertainty surrounding the perioperative management of direct oral anticoagulants (DOACs). DOACs are becoming more commonly used, with its ease of use, predictable pharmacokinetic profile and lack of monitoring requirements as compared to warfarin. Early studies have reported that surgeries were significantly delayed for DOAC patients and attributed the delay to the uncertainties in the perioperative management of these drugs^17,18^. Existing protocols are rather ambiguous and current practices are largely based on experts’ opinions. DOACs have a half-life of up to 15 hours depending on renal function^19^. Older age is a key predictor of chronic kidney disease^20^ and with age-related decline in renal function^21^, this poses a concern especially in elderly patients as they may require to hold off DOACs up to 96 hours prior to surgery^19^. Antidotes are currently being developed, however are largely licensed for life-threatening bleeds and must be weighed against the risk of thrombosis. Cost may also be a limiting factor^22^. Hence, surgical delay remains as the only option to allow for drug washout. The concerns around DOAC use, similar to clopidogrel, are bleeding tendencies.

The purpose of our study is to evaluate the impact of preoperative use of antiplatelets or anticoagulants on the time to surgery (TTS) in elderly hip fracture patients in Singapore as well as one-year mortality rates. To our knowledge, there has been no such study conducted in the Asian context despite the prevalent use of antiplatelets and anticoagulants in the Singaporean population.

## Materials and Methods

We conducted a single-centred retrospective cross-sectional study on all hip fracture patients admitted to the orthogeriatric unit of an acute care public hospital serving the Eastern region of Singapore over a 12-month period between 01 November 2016 to 31 October 2017. Inclusion criteria included age 60 years and above, having sustained a low energy trauma such as falling from standing height, sustained a neck of femur, intertrochanteric or subtrochanteric fracture and having undergone surgical fixation of hip fracture. Patients were excluded if they were younger than 60 years old, had non-operative management of fracture, having sustained multiple acute fractures at different sites, high impact trauma, pathological fractures and peri-prosthetic fractures. Patients in this dedicated unit were managed with evidence-based best practices in multi-disciplinary care model^23^.

This study was approved by Singhealth Centralised Institutional Review Board (CIRB Ref: 2018/3145) and waiver of consent was granted for the purpose of this study. The following variables were extracted from the database including: basic demographic data, patient comorbidities which includes heart failure, asthma, chronic obstructive pulmonary disease (COPD), diabetes, hypertension, hyperlipidemia, ischemic heart disease (IHD), peripheral vascular disease (PVD), renal failure and dementia, pre-operative American Society of Anesthesiologist (ASA) score, premorbid Parker mobility (PM) score, premorbid modified Barthel Index (MBI) score, type of fracture, admission haemoglobin (Hb), length of stay, TTS and one-year mortality. Reasons for surgical delays and inpatient complications were also recorded on our dataset.

**Fig. 1:**
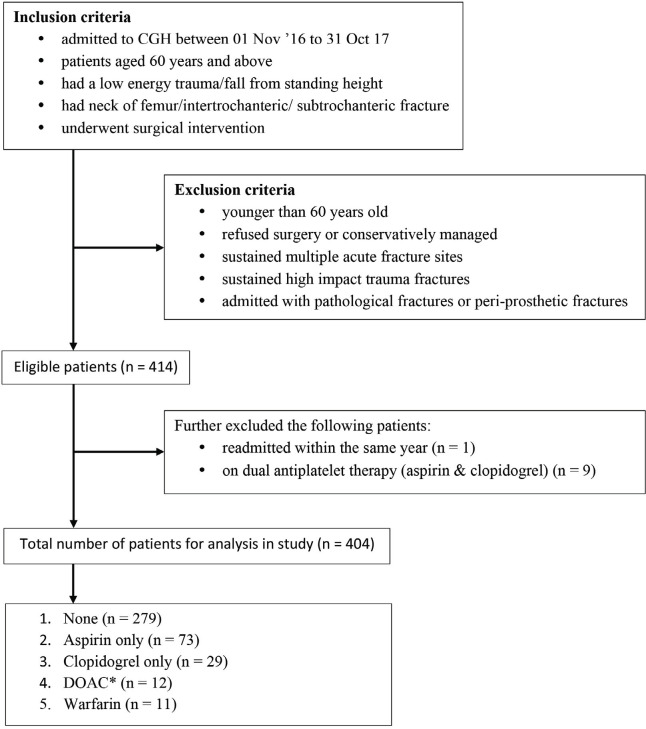
Flowchart of patient recruitment into study.

Following a review of patient’s individual medical records, premorbid use of antiplatelets (e.g. aspirin, clopidogrel) and anticoagulants (e.g. warfarin, apixaban, dabigatran or rivaroxaban) were obtained from patient’s pre-admission medication list and recorded alongside respective indications. Patients were assigned into five different groups depending on type of antiplatelets or anticoagulants. Patients who were on dual antiplatelets were excluded from the study to avoid any confounding results when comparing the outcomes between different pharmacological groups (Fig. 1).

Abbreviation - * DOAC: Direct Oral Anticoagulant (includes apixaban, dabigatran, rivaroxaban)

The primary outcome measure for this study was TTS, defined as time from inpatient admission to surgery and further categorised into two different groups, (i) early surgery (within 48 hours), and (ii) delayed surgery (more than 48 hours). One-year mortality rates were also analysed as a secondary outcome.

Categorical data were presented as number (percentage) and continuous data as mean ± standard deviation (SD) unless otherwise stated. Differences in patient characteristics among categories of medication (aspirin, clopidogrel, warfarin, DOACS and none) were studied using chi-square test for categorical variables or one-way ANOVA for continuous variables with pairwise comparison. Cox proportional hazards model was used to assess risk of one-year mortality^24^. The models were adjusted for age, gender, race, premorbid PM score, premorbid MBI score, premorbid AMT score, number of co-morbidities, number of inpatient complications, type of hip fracture and ASA status. Statistical significance was taken at p<0.05. Statistical analysis was performed using STATA version 14 [STATA Corporation, College Station, Texas].

## Results

A total of 414 consecutive hip fracture admissions were enrolled in this study based on the defined inclusion and exclusion criteria. Ten patients had to be further excluded from the study, as one patient was readmitted within the same study period, and nine who were on dual antiplatelet therapy. Out of 404 patients, 73 (18%) patients were on aspirin, 29 (7%) on clopidogrel, 12 (3%) on DOACs and 11 (3%) on warfarin. The rest constituted the control group who were not on any antiplatelets nor anticoagulants prior to admission (n=279,69%).

Patients’ baseline characteristics are presented in Table I while the summary of use of antiplatelets and anticoagulants in this study cohort is presented in Table II. Table I demonstrates that there were no significant gender or ethnic differences when comparing data across the five groups with majority of patients admitted were female (68%). Patients in DOAC group were significantly older than clopidogrel patients (p=0.018).

**Table I:** Patients’ baseline characteristics.

	None (n=279)	Aspirin (n=73)	Clopidogre (n=29)	DOACs (n=12)	Warfarin (n=11)
Gender, n (%)					
Female	200 (71.7)	46 (63)	17 (58.6)	8 (66.7)	5 (45.5)
Age, mean ± SD	80.6 ± 86	81.4 ± 7.8	78.7 ± 8.3	85.4 ± 5.5^a^	79 ± 6.0
Race					
Chinese, n (%)	222 (79.6)	56 (76.7)	21 (72.4)	10 (83.3)	8 (72.7)
Malay, n (%)	33 (11.8)	10 (13.7)	5 (17.2)	1 (8.3)	1 (9.1)
Indian, n (%)	7 (2.5)	2 (2.7)	0	0	0
Others, n (%)	17 (6.1)	5 (6.9)	3 (10.3)	1 (8.3)	2 (18.2)
ASA score					
1-2, n (%)	98 (35)	5 (7)	5 (17)	2 (17)	0
3-4, n (%) Premorbid PM score	181 (65)	68 (93)	24 (83)	10 (83)	11 (100)
Mean ± SD	5.67 ± 3.08	4.5 ± 3.95^b^	4.68 ± 3.09	3.55 ± 2.91^c^	6.6 ± 3.20
Premorbid MBI score, Mean ± SD	70.9 ± 25.2	61.5 ± 28.2^d^	72.6 ± 23.9	50.9 ± 34.9^e^	53.9 ± 29.0^f^
AMT score, Mean ± SD	6.71 ± 3.30	6.33 ± 3.11	6.66 ± 3.31	4.33 ± 3.85^g^	8.10 ± 2.88
Admission Hb, (g/dL) Mean ± SD	11.8 ± 1.86	11.8 ± 1.70	11.8 ± 1.72	11.7 ± 1.69	11.0 ± 1.08
Type of fracture					
NOF, n (%)	139 (49.8)	28 (38.4)	15 (51.7)	5 (41.7)	5 (45.5)
IT, n (%)	132 (47.3)	44 (60.3)	14 (48.3)	6 (50.0)	6 (54.5)
Subtrochanteric, n (%)	8 (2.9)	1 (1.3)	0	1 (8.3)	0
No. of comorbidities	1.72 ± 1.17	2.86 ± 1.08^h^	2.74 ± 1.43^h^	2.83 ± 1.27^h^	3.0 ± 1.26^h^

Abbreviations – AMT: Altered Mental Status, ASA: American Society of Anesthesiologists, Hb: Haemoglobin, IT: Intertrochanteric, MBI: Modified Barthel Index, NOF: Neck of Femur, PM: Parker’s mobility

Notes: ^a^: DOAC patients are older than clopidogrel patients by 6.76 years (p = 0.018). ^b^: Aspirin patients had poorer premorbid Parkers score by 0.11 compared to None group (p= 0.014). ^c^: DOAC patients had poorer premorbid Parkers score by 2.12 compared to None group (p = 0.026) & poorer premorbid Parkers score by 3.05 compared to warfarin (p=0.024). ^d^: Aspirin patients had lower premorbid MBI score by 9.39 compared to None (p= 0.007). ^e^: DOAC patients had lower premorbid MBI score by 20.0 compared to None (p=0.010) & lower MBI score by 21.7 compared to clopidogrel (p=0.016). ^f^: Warfarin patients had lower premorbid MBI score by 17.0 compared to None (p=0.035) and lower premorbid score by 18.7 compared to clopidogrel (p=0.044). ^g^: DOAC patients had lower AMT score on admission by 2.38 compared to None (p=0.014), lower by 2.32 compared to clopidogrel (p=0.034) & lower by 3.77 compared to warfarin (p=0.007). ^h^: All pharmacological groups have significantly higher number of comorbidities compared to None. Aspirin has more comorbidities by 1.14 (p < 0.001), clopidogrel has 1.02 more (p < 0.001), warfarin has 1.28 more (p < 0.001) and DOAC has more comorbidities by 1.11 (p = 0.002) as compared to None.

**Table II:** Antiplatelet and anticoagulant use and its indication.

**Type of antiplatelet**	**(N=102)**	**Indication of antiplatelets**	
Aspirin	73	IHD	45
Clopidogrel	29	CVA	33
		AF	4
		PVD	3
		Others/Unknown	17
**Type of anticoagulant**	**(N=23)**	**Indication of anticoagulants**	
Apixaban	5	AF	22
Dabigatran	2	PVD	1
Rivaroxaban	5		
Warfarin	11		

Abbreviations – AF: Atrial Fibrillation, CVA: Cardiovascular Accident, IHD: Ischemic Heart Disease, PVD: Peripheral Vascular Disease

Comorbidities which include heart failure, asthma, chronic obstructive pulmonary disease (COPD), diabetes, hypertension, hyperlipidemia, ischemic heart disease (IHD), peripheral vascular disease (PVD), renal failure and dementia, were analysed as cumulative number of comorbidities for each patient. All of the pharmacological groups had significantly higher number of comorbidities compared to the group without. There were no significant differences in number of comorbidities when comparing between aspirin, clopidogrel, DOAC and warfarin groups. Admission haemoglobin (Hb) levels across the five different pharmacological groups did not differ significantly (p=0.537).

**Fig. 2:**
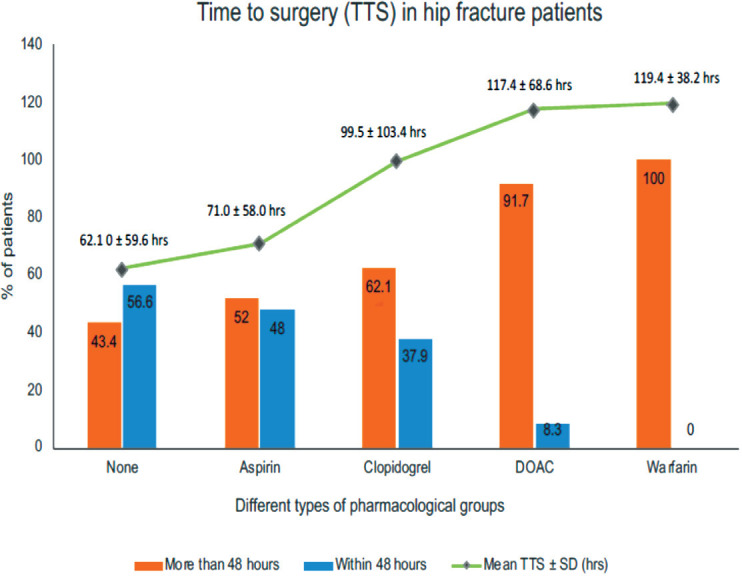
Chart illustrating the percentage of patients experiencing a surgical delay (>48 hrs) and mean time to surgery in each group.

Comparing the mean TTS in each group, patients on warfarin underwent surgery later than other groups (mean TTS 119.4hr ± 38.2) (Fig. 2). Percentages of patients in each group whom experienced an early or delayed surgery is demonstrated in Fig. 2.

A logistic regression analysis was carried out to determine the association between medications use and surgical delay (Table III). The analysis was adjusted for other factors that could influence TTS including age, gender, race, premorbid PM, MBI, AMT and ASA scores, type of fracture and number of comorbidities. All patients who were taking warfarin had delayed surgery, hence no values were reported for these patients. After adjusting for the factors above, we found that patients who were on clopidogrel (p<0.001) and DOACs (p=0.001) were more likely to have delayed surgery as compared to patients who were not on any antiplatelets or anticoagulants.

**Table III:** Time to surgery beyond 48hours after adjusting for the following factors.

Time to surgery > 48hrs (hrs)	Odds Ratio	Std Error	Z	p-value	95% confidence	interval
Aspirin	0.508	0.365	-0.94	0.346	0.124	2.08
Clopidogrel	23.2	17.9	4.08	0.000	5.11	105.1
DOACs	93.0	132.6	3.18	0.001	5.67	1523.0
Warfarin			1 (empty)*			
Age	0.994	0.033	-0.17	0.863	0.932	1.06
Gender, male	1.15	0.730	0.22	0.823	0.333	3.99
Chinese	1.73	1.30	0.73	0.466	0.397	7.53
Malay	2.44	2.23	0.97	0.330	0.405	14.7
Indian			1 (empty)*			
Premorbid Parkers Score	0.924	0.093	-0.79	0.431	0.758	1.13
Premorbid MBI score	1.01	0.011	0.76	0.447	0.987	1.03
No. of other reasons for delay	374.1	253.4	8.75	0.000	99.2	1410.9
No. of comorbidities	1.09	0.210	0.44	0.661	0.746	1.59
IT #	0.895	0.445	-0.22	0.823	0.337	2.37
Subtrochanteric #	0.872	1.76	-0.07	0.946	0.017	45.8
ASA, 3-4	0.681	0.429	-0.61	0.542	0.198	2.34
AMT score	1.10	0.115	0.91	0.363	0.897	1.35

Abbreviations - #: fracture, AMT: Altered Mental Status, ASA: American Society of Anesthesiologists, IT: Intertrochanteric, MBI: Modified Barthel Index.

Note: * Values are empty as all Warfarin and Indian patients had delayed surgery, beyond 48 hours.

The actual recorded reasons for delayed surgery are listed in Table IV. Number of additional reasons for delayed surgery was also incorporated in the multivariate analysis and found that with increasing number of additional reasons for delayed surgery, patients were more likely to have delayed surgery (p<0.001).

**Table IV:** Reasons for delayed surgery beyond 48 hours.

Reasons for surgical delay beyond 48 hrs*	n (% of total no. of reasons)
Use of anticoagulants/antiplatelets	14 (5.7)
System-related issues	55 (22.4)
Awaiting patient & family decision	40 (16.3)
Medically unfit	45 (18.4)
Pending investigations	42 (17.1)
Referral to other disciplines (CVM, Anaesthesia, Others)	46 (18.8)
Unknown	3 (1.2)

Note: * Each patient can have more than 1 reason for surgical delay.

**Fig. 3:**
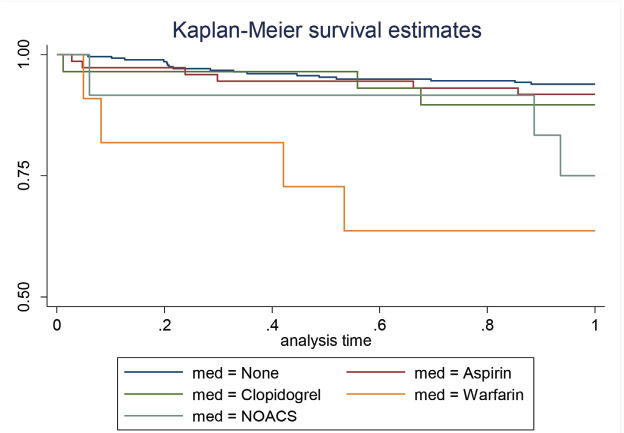
Kaplan-Meier survival curve to show the effect of different pharmacological groups on mortality rates.

This was analysed as a secondary outcome of our study. Patients who were on warfarin had the highest one-year mortality rate of 36.4% when compared to other pharmacological groups (Table V). Patients who were on warfarin were 10 times (p=0.001) more likely to die within a year (Table VI). This was further demonstrated on the Kaplan-Meier survival curve (Fig. 3) whereby patients who were on warfarin had lower one year survival rates as compared to the other agents.

**Table VI:** Association between patients’ baseline characteristics and one-year mortality rates.

One- year mortality rate	Haz. Ratio	Std Error	Z	P	95% confidence	interval
Aspirin	0.805	0.487	-0.36	0.720	0.246	2.63
Clopidogrel	1.81	1.22	0.88	0.381	0.481	6.78
DOACs	3.24	2.51	1.51	0.129	0.710	14.8
Warfarin	10.4	7.54	3.21	0.001	2.52	43.1
Age	1.01	0.028	0.22	0.829	0.953	1.06
Gender, Male	1.79	0.758	1.37	0.17	0.778	4.10
Chinese	1.88	1.58	0.75	0.456	0.359	9.80
Malay	2.33	2.23	0.88	0.379	0.355	9.79
Indian	14.3	15.2	2.52	0.012	1.80	114.0
Premorbid Parkers Score	0.851	0.069	-1.99	0.047	0.726	0.998
Premorbid MBI score	1.01	0.008	0.84	0.399	0.991	1.02
No. of inpatient complications	1.50	0.354	1.71	0.087	0.944	2.38
No. of comorbidities	1.06	0.176	0.32	0.745	0.762	1.46
IT #	1.24	0.539	0.48	0.628	0.525	2.91

Abbreviations - #: fracture, DOAC: Direct Oral Anticoagulant, IT: Intertrochanteric, MBI: Modified Barthel Index

## Discussion

The discussion revolving around optimal timing for surgery mainly concerns the association between surgical delay and perioperative mortality rates. Our institution’s best practice guidelines target surgery for patients with hip fractures within 48 hours of admission. However, surgical delay can occur due to numerous factors, including premorbid antiplatelets or anticoagulants use. This has posed a clinical dilemma for physicians to balance between a higher bleeding risk with an early operation versus a surgical delay. In our study, we found a significant association between the use of clopidogrel (p<0.001) and DOACs (p=0.001) with surgical delay even after accounting for other possible confounding factors. Prior studies have suggested that patients on antiplatelets and anticoagulants inherently have a higher number of comorbidities, resulting in longer time for medical optimisation, however our study did not find this to be the case. Number of comorbidities was not a significant predictor of surgical delay as illustrated in this study.

Earlier studies have demonstrated greater significant drop in mean Hb level and perioperative blood loss in clopidogrel users with early surgical intervention within 48 hours than those delayed^25,26^. However, more recent studies have proven that despite undergoing early surgery^27^ or without clopidogrel withdrawal pre-operatively^28^, clopidogrel patients did not have significantly worse outcomes in relation to blood-loss or mortality rates^29^. They argued that given hip fracture itself could promote a prothrombotic state, cessation of clopidogrel raises concerns of risk for cardiovascular events. One study demonstrated a 20.2% increase in acute coronary syndrome (ACS) events in hip fracture patients with their clopidogrel withheld, peaked at 4-8 days after the antiplatelet withdrawal^30^. Overall, these results complement our findings where clopidogrel users did not experience any significant differences in one-year mortality rate. Despite increasing evidence of clopidogrel continuation perioperatively, there is still much variation to the practice on grounds, largely based on orthopaedic surgeon’s decisions^31^.

DOACs use has also been implicated in causing significant surgical delays, with up to three times longer than non-anticoagulated patients^32^. Similarly, to our study, the median TTS for DOAC patients was almost double compared to none group. Uncertainties regarding perioperative management and bleeding risk were amongst the reasons for surgical delay^17^. Other studies have contended whether DOAC use has resulted in unnecessary surgical delay, given that despite absence of anticoagulation reversal, DOAC patients did not experience greater blood loss, in-hospital complications nor mortality^33,34^. In contrast, Leer and colleagues did not find DOACs use to influence surgical delay, but this could be due to choice anaesthesia, where half of DOAC patients in the study received general anaesthesia^35^. Anaesthesia experts have also urged clinicians to focus more on risk of delay and thromboembolic events and suggested expediting surgery by considering appropriate anaesthesia methods in hip fracture DOAC users^36^.

All warfarin patients in this study experienced significant surgical delay, with almost a wait of 5 days before surgery. Previous studies in the past focused mainly on INR reversal strategies in order to minimise surgical delay^37,38^. However, it has been demonstrated that INR reversal may not be the key reason since warfarin users still experienced delay despite the presence of reversal agents or presenting with appropriate INR. Surgical delay was mainly attributed to medical reasons including cardiac or pulmonary assessment or due to an ongoing infection^18,39^. Similarly, in our study, warfarin patients presented with highest number of comorbidities with significantly lower premorbid MBI score suggesting that these patients are likely to be more dependent in their activities of daily living (ADLs), leading to possibly poorer patient outcomes. We also found that warfarin patients had poorer survival with the highest one-year mortality rate of 36.4% as compared the other groups. This mortality finding should be interpreted with caution given the small number of patients on warfarin. In a recent study, 54% of warfarin patients who had delayed surgery experienced a significantly higher median blood loss and higher mortality rates within one-year post-operation than other groups^40^. Hence, hip fracture patients on warfarin are at high risk for delay and mortality, requiring special attention for medical and INR optimisation with necessary timely investigations in order for safe early surgery.

Therefore, it is evident that apart from periprocedural management of antiplatelets and anticoagulants which focuses on drug reversal, medical optimisation of patients’ conditions needs to be carried out concurrently given the complexity of patients’ profile. The results from this study provides better evidence-based data in guiding surgeons’ decisions and streamline the management of hip fracture patients presenting with premorbid use of antiplatelets or anticoagulants in the future, leading to enhancement of existing in-house hip fracture protocols. Interdisciplinary inputs of the orthopaedic surgeon, anaesthetist, geriatrician and pharmacist are essential in establishing these protocols, whilst individualised team-based decision-making on timing of surgery considering the unique profile of each individual should be the way forward.

To our best knowledge, this is the first study conducted in Singapore and within the Southeast Asia region in evaluating the effect of both antiplatelets and anticoagulants use on TTS in hip fracture patients, confirming that the impact on delaying surgery is a significant issue to address. Our study also provides a comprehensive analysis by including both broad pharmacological groups of antiplatelets and anticoagulants utilising a multivariate analysis by adjusting for all other possible confounders. It provides impetus for regional hip fracture teams, which include orthopaedic surgeons, anaesthetists, geriatricians and pharmacists to develop or enhance protocols for patients on these medications to achieve early surgery without compromising medical optimisation.

This study however has its limitations. Given the retrospective nature of the study, we are unable to determine a direct cause and effect relationship between use of antiplatelets and anticoagulants to the study outcomes. This study was also conducted between year 2016 to 2017, of which there could have been changes to current practice guidelines at present. Moreover, the use of these medications are likely also linked to underlying vascular comorbidities for which there may be confounding reasons for operative delay. Despite recruiting a substantial number of patients, the numbers in each medication group is small, which could have underpowered the study in detecting more differences in study outcomes. Additionally, possible acute cardiovascular or thromboembolic complications arising from the suspension of these medications were not captured nor analysed. Lastly, this was a single-centred study which might affect the ability to generalise the findings across the region, given the heterogeneity of clinician opinions and variability of hip fracture protocols across various healthcare institutions. Future larger prospective multi-centred studies may be helpful to determine the causal effects of the use of antiplatelets and anticoagulants on TTS and clinical outcomes of hip fracture patients.

## Conclusion

In conclusion, this study has established how premorbid use of clopidogrel, and anticoagulants are significant predictors of surgical delay in hip fracture patients in our local population. Surgical delay negatively impacts patients’ outcomes, and we continue to advocate for an early surgery, within 48 hours for all hip fracture patients who have been medically optimised.
